# Supplementary material for: Detecting the PEX Like Domain of Matrix Metalloproteinase-14 (MMP-14) with Therapeutic Conjugated CNTs
Source: Biosensors (Basel). 2022 Oct 17;12(10):884. doi: 10.3390/bios12100884 (PMC9599479; doi:10.3390/bios12100884)
Supplement: Supplementary file 1 [file biosensors-12-00884-s001.zip › biosensors-1885985-supplementary.pdf]

Detecting the PEX like domain of Matrix Metalloproteinase-14 (MMP-14) with therapeutic conjugated CNTs

D. Vieira[1], J. Barralet[1,2], E. Harvey[1,2], G. Merle[1,3]*

[1] McGill University, Faculty of Medicine, Department of Experimental Surgery

[2] Department of Surgery, McGill University, Montreal, Canada

[3] Polytechnique Montreal, Department of Chemical Engineering

**Corresponding author*

Supplementary information

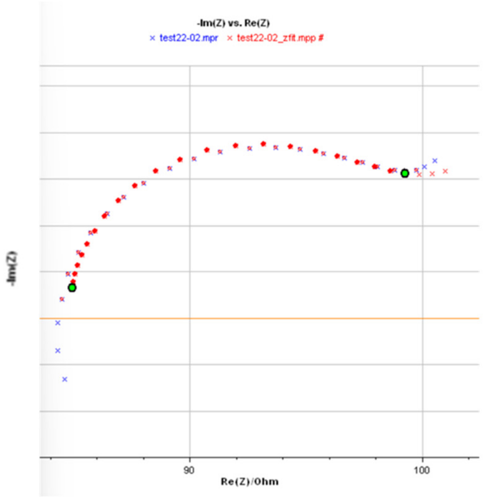

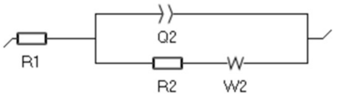

Parameter	Value	Unit	Deviation
R1	84.8	Ohm	1
Q2	2.422×10^{-6}	$F.s^{(a-1)}$	1
a2	1		0.5
R2	13.21	Ohm	0.8544
s2	244.8	$Ohm.s^{-1/2}$	0.01775
χ^2	0.112×10^{-9}	-	-

Figure S1 – Circuit fit for inhibitor loaded CNT electrode after interaction with 50 ng.ml^{-1} of MMP-14 in PBS (pH 7.40) containing 10 mmol.ml^{-1} of $K_4[Fe(CN)_6]^-$, 10 mmol ml^{-1} of $K_3[Fe(CN)_6]^-$ and 10 mmol.ml^{-1} of NaCl, applied potential of +0.20V, from 50kHz to 500Hz, amplitude 50 mV.

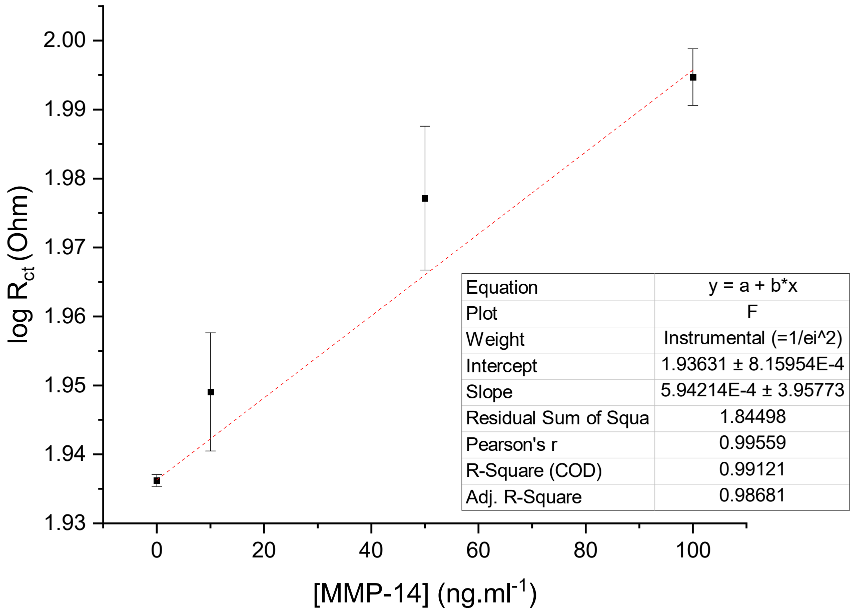

Figure S2 – Linear fit of log Rct response for different concentrations of MMP-14. Protein concentration varying from 10 ng.mL-1 to 100 ng.mL-1, applied potential of +0.20V, from 50kHz to 500Hz, amplitude 50 mV

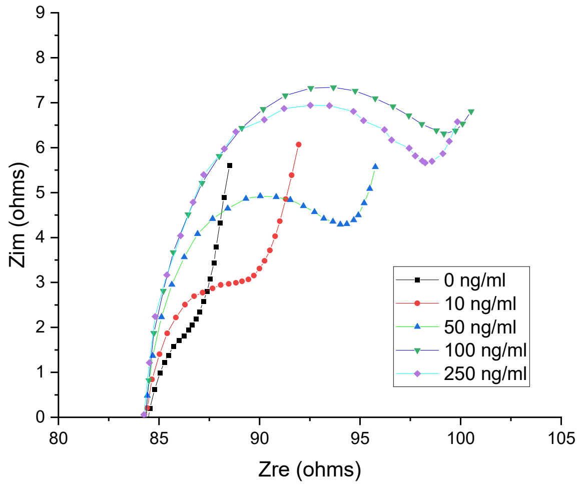

Figure S3 – EIS response of inhibitor loaded CNT after interaction with MMP-14, varying concentration from 0 to 250 ng.ml^{-1}, in PBS (pH 7.40) containing 10 mmol.ml^{-1} of $K_4[Fe(CN)_6]^-$, 10 mmol ml^{-1} of $K_3[Fe(CN)_6]^-$ and 10 mmol.ml^{-1} of NaCl (applied potential of +0.20V, from 50kHz to 500Hz, amplitude 50 mV).

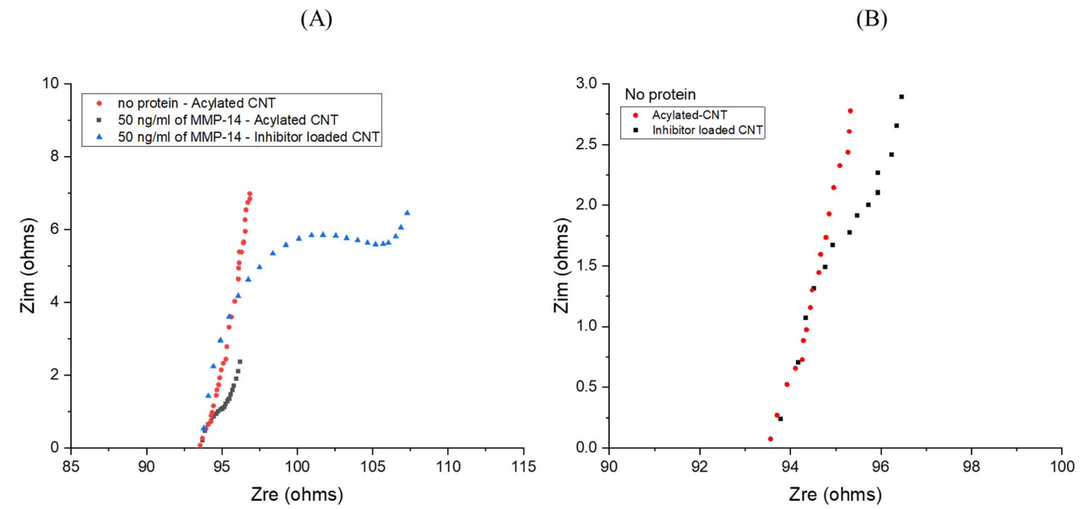

Figure S4 – (A) EIS response of acylated CNT after interaction with 50 ng.ml^{-1} of MMP-14 and (B) EIS response in absence of protein for acylated and inhibitor loaded CNT in PBS (pH 7.40) containing 10 mmol.ml^{-1} of $K_4[Fe(CN)_6]^-$, 10 mmol ml^{-1} of $K_3[Fe(CN)_6]^-$ and 10 mmol.ml^{-1} of NaCl, applied potential of +0.20V, from 50kHz to 500Hz, amplitude 50 mV.
